# Revisiting the evolutionary trend toward the mammalian lower jaw in non-mammalian synapsids in a phylogenetic context

**DOI:** 10.7717/peerj.15575

**Published:** 2023-06-20

**Authors:** Tomohiro Harano, Masakazu Asahara

**Affiliations:** Division of Liberal Arts and Sciences, Aichi Gakuin University, Nisshin, Aichi, Japan

**Keywords:** Synapsida, Therapsida, Cynodontia, Dentary

## Abstract

The mammalian lower jaw comprises a single bone, the dentary, which is a unique feature among vertebrates. The lower jaws of extinct non-mammalian synapsids were composed of the dentary and several postdentary bones. Synapsid fossils exhibit variation in the dentary size relative to the overall lower jaw. An evolutionary trend toward dentary enlargement and postdentary reduction in non-mammalian synapsids has long been documented but has not been established using modern phylogenetic comparative methods. In this study, we examine the evolutionary pattern of dentary size relative to the lower jaw through phylogenetic analyses of measurements in a broad range of non-mammalian synapsid taxa. Our analyses revealed an evolutionary trend toward dentary area enlargement relative to the overall lower jaw in the lateral view across all non-mammalian synapsids. This trend is likely due to vertical expansion of the dentary given that the same trend is not evident when looking at anterior to posterior measurements of the dentary relative to the lower jaw as a whole in lateral view. Ancestral character reconstructions revealed that the evolution of the measurements was not unidirectional in non-mammalian synapsids. Our results provide no evidence of an evolutionary trend toward the dentary enlargement at the expense of postdentary bones across non-mammalian synapsids. This implies that the evolutionary origin of the mammalian lower jaw is not adequately explained by the evolutionary trend of dentary enlargement throughout non-mammalian synapsids. Instead, selection that occurred during the transition from non-mammalian cynodonts to early mammals may have produced the mammalian lower jaw.

## Introduction

Mammals are characterized by a lower jaw comprising a single bone: the dentary (Feldhamer et al., 2003; Sidor, 2003; Kemp, 2005). In contrast, the lower jaws of non-mammalian vertebrates comprise several bones (Sidor, 2003; Kemp, 2005). Mammals and other extant amniotes, such as reptiles and birds, diverged more than 320 million years ago and have separately evolved since then; therefore, extinct non-mammalian synapsids can provide useful information about the evolution of such mammalian features (Jones et al., 2018). As an ancestral condition, non-mammalian synapsids had a lower jaw composed of the dentary and up to seven postdentary bones articulated to the cranium through the jaw joint between the articular and quadrate (Sidor, 2003; Navarro-Díaz, Esteve-Altava & Rasskin-Gutman, 2019). Fossil records indicate gradual dentary enlargement and the reduction of the postdentary bones in the lower jaw during the transition from non-mammalian synapsids to mammals (Barghusen & Hopson, 1970; Feldhamer et al., 2003; Sidor, 2003; Kemp, 2005; Kemp, 2016; Angielczyk & Kammerer, 2018; Navarro-Díaz, Esteve-Altava & Rasskin-Gutman, 2019). In early mammals, some of the postdentary bones were lost, and some of the bones forming the ancestral reptilian jaw joint (*i.e.,* articular, angular, and quadrate bones) were incorporated into the mammalian middle ear (*i.e.,* malleus, ectotympanic, and incus); this led to the emergence of the novel mammalian jaw joint between the dentary and squamosal bones (Barghusen & Hopson, 1970; Feldhamer et al., 2003; Sidor, 2003; Kemp, 2005; Kemp, 2016; Luo, 2011; Luo, Schultz & Ekdale, 2016; Lautenschlager et al., 2017; Tucker, 2017; Angielczyk & Kammerer, 2018; Navarro-Díaz, Esteve-Altava & Rasskin-Gutman, 2019). The novel mammalian jaw joint allowed for precise occlusion between the upper and lower teeth, consequently enabling efficient mastication, and increased resistance to jaw dislocation (Kemp, 2006; Tucker, 2017; Navarro-Díaz, Esteve-Altava & Rasskin-Gutman, 2019). In addition, the incorporation of multiple bony elements into the middle ear increased sensitivity to high-frequency sounds (Allin, 1975; Luo, 2011; Kemp, 2016; Luo, Schultz & Ekdale, 2016).

Synapsida includes Therapsida, which in turn includes Cynodontia. Within Cynodontia, Mammaliamorpha includes Mammaliaformes, comprising all mammals and their closest extinct relatives, such as *Morganucodon* (Rowe, 1988). Each of these taxa represents a subclade within synapsids. Non-mammalian synapsids first appeared approximately 310–320 million years ago (late Carboniferous period) (Angielczyk & Kammerer, 2018). During approximately 150 million years of their history, non-mammalian synapsids exhibited extremely high morphological diversity (Sidor, 2001; Kemp, 2005). Late-diverging non-mammalian synapsids exhibited a larger dentary size relative to the overall lower jaw compared with early synapsids, a pattern that has been documented for a long time (Broom, 1904). A trend toward a relatively large dentary, representing a mammal-like condition, has been considered in the context of synapsid phylogeny (Olson, 1944; Angielczyk & Kammerer, 2018). Sidor (2003) used a phylogeny reconstructed through numerical cladistic analyses and conducted a quantitative examination to reveal a positive correlation between the relative dentary size and both stratigraphic and phylogenetic positions across Synapsida and the subclades encompassing Mammaliaformes, providing support for a gradual trend of increasing relative dentary size throughout the course of synapsid evolution. However, such correlations are not apparent in the subclades that do not include Mammaliaformes; therefore, a consistent increase in the relative dentary size is not universal to all synapsid subclades (Sidor, 2003).

Body size reduced during the transition from early cynodonts to mammaliaforms (Sookias, Butler & Benson, 2012; Lautenschlager et al., 2018). Biomechanical analyses suggest that this miniaturization was a factor in the evolution of the jaw joint in early mammals (Lautenschlager et al., 2018). A smaller structure can hold fewer elements (Sidor, 2001); thus, miniaturization may be associated with the reduction of the postdentary bones (Sidor, 2003). However, Sidor (2003) showed that the dentary and postdentary areas scale nearly isometrically with overall jaw length across synapsids, suggesting that miniaturization is not sufficient to explain postdentary reduction.

To the best of our knowledge, no previous studies have employed modern phylogenetic comparative methods to investigate an evolutionary trend of dentary enlargement and postdentary reduction in non-mammalian synapsids. Recently, a time-calibrated comprehensive phylogeny containing numerous synapsid taxa was reconstructed and comparative studies were conducted (Jones, Angielczyk & Pierce, 2019; Jones et al., 2021). The general approach uses a Brownian motion (BM) process as a standard model for trait evolution on a phylogeny, which assumes that the trait changes randomly in magnitude and direction and gradually through time (Felsenstein, 1985). A directional trend across phylogeny of the trait value either increasing or decreasing over time can be described using a trend model that incorporates the trend parameter into the BM model, thus allowing for statistical evaluation of the effect of the trend on trait evolution (Pagel, 1999; Pagel, 2002).

In the present study, we examined the evolution of the dentary size relative to the overall lower jaw in non-mammalian synapsids through phylogenetic comparative analyses. We reconstructed the evolutionary history of this trait and attempted to determine whether there is an evolutionary trend of dentary enlargement across non-mammalian synapsids. We also tested the fit of a series of evolutionary models to characterize the evolutionary pattern of the relative size of the dentary in non-mammalian synapsids. In a previous study, Sidor (2003) used an index that summarized the area and linear measurements of the dentary in the lateral view of the lower jaw to quantify the relative contribution of the dentary to the lower jaw in synapsids. As a simple measure of the relative contribution of the dentary to the lower jaw, we used the area of the dentary relative to the total area of the lower jaw in the lateral view. This measure corresponds to the size of the dentary relative to the postdentary bones in the lower jaw, although it can increase without a reduction in the postdentary bones if the dentary expands vertically. To evaluate the dentary enlargement at the expense of the postdentary bones, we used the anteroposterior distance from the anterior end of the lower jaw to the posterior boundary of the dentary region (hereafter referred to as dentary length) relative to the total length of the lower jaw in the lateral view as another measure.

## Materials & Methods

### Measurements

Illustrations of the lateral views of lower jaws taken from the literature (Dataset S1) were used to obtain the area and length of the dentary and lower jaw. The illustrations may simplify certain aspects of the actual specimens, which could result in errors in measurements. While we assumed that the effects of these errors would be negligible in our analyses, they cannot be completely eliminated. Measurements of morphological traits obtained from illustrations were included in some previous comparative studies (*e.g.*,  Slater & Van Valkenburgh, 2008; Church et al., 2019; Harano & Asahara, 2022). The illustrations in our sample were taken from 53 taxa of non-mammalian synapsids, which were identified at the species level if possible, or at the genus level if the species was not identified. Of the 53 taxa, 41 belonged to Therapsida, which included Biarmosuchia, Dinocephalia, Anomodontia, Gorgonopsia, Therocephalia, and Cynodontia.

**Figure 1:**
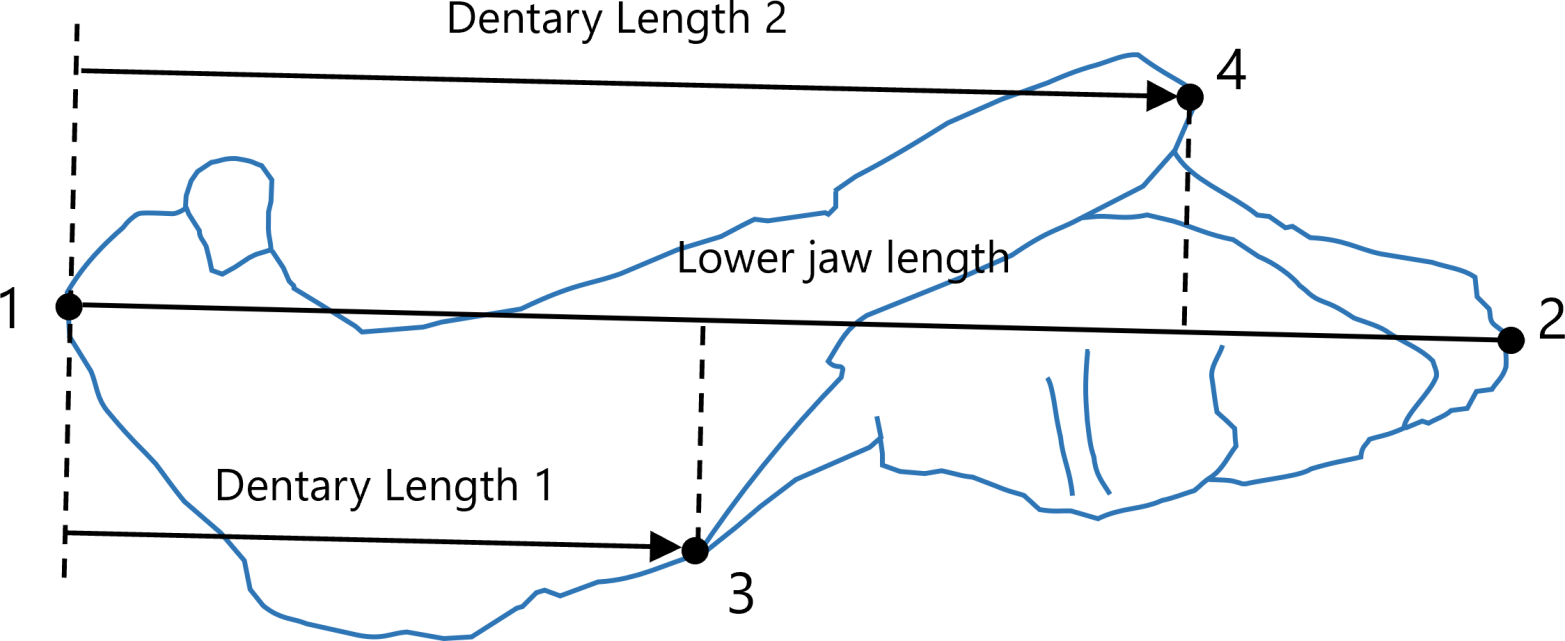
Positions of landmarks (closed circles) in the lateral view of the lower jaw. Landmarks were defined as follows: 1, most anterior point on the lower jaw; 2, most posterior point on the lower jaw; 3, anterior end of contact between the dentary and the postdentary regions excluding the splenial; 4, most posterior point on the dentary. Lower jaw length was calculated as the distance from landmark 1 to landmark 2. The intersections of the line connecting landmark 1 to landmark 2 with a perpendicular line (denoted by dotted lines) from landmarks 3 and 4 to the line connecting landmark 1 to landmark2 were used to determine the anteroposterior positions of landmarks 3 and 4. Dentary Length 1 was calculated as the distance from landmark 1 to the anteroposterior position of landmark 3, whereas Dentary Length 2 was calculated as the distance from landmark 1 to the anteroposterior position of landmark 4. The diagram of the lower jaw used in this representation was drawn based on an illustration of *Leontosaurus vanderhorsti* in Kammerer (2016).

The areas of the dentary and the total lower jaw were measured using ImageJ version 1.53 (Schneider, Rasband & Eliceiri, 2012). However, the areas of 10 taxa in our sample were not measured as the lower jaw was partially obscured by the cranium in the available illustrations. Consequently, data on these areas were obtained for 43 taxa. To evaluate the lengths of the dentary and total lower jaw, four landmarks were digitized onto the illustrations using tpsDig version 2.31 (Rohlf, 2017), the configuration of which is shown in Fig. 1. The total length of the lower jaw was measured as the distance from the anterior end to the posterior end of the lower jaw (the distance from landmark 1 to landmark 2 in Fig. 1). Two measurements of the dentary length were taken: Dentary Length 1 was defined as the distance from the anterior end of the lower jaw (the anterior end of the dentary; landmark 1 in Fig. 1) to the anteroposterior position of the anterior end of contact between the dentary and the postdentary regions, excluding the splenial (landmark 3 in Fig. 1); Dentary Length 2 was defined as the distance from the anterior end of the lower jaw (landmark 1 in Fig. 1) to the anteroposterior position of the posterior end of the dentary region (landmark 4 in Fig. 1). These anteroposterior positions were determined along the line connecting the anterior to the posterior end of the lower jaw (line connecting landmark 1 to landmark 2 in Fig. 1), as shown in Fig. 1. If the illustration did not include a scale, available data on the length of the skull or lower jaw of the taxon were used as a proxy to allow for the conversion of the areas and lengths seen in the illustration into an actual measurement usable in the analysis. The measurements for all taxa included in this study are presented in Dataset S2. All data were converted to natural logarithms prior to subsequent analyses.

### Phylogenetic comparative analyses

The phylogeny of synapsids used in this study was derived from the supertree reconstructed by Jones, Angielczyk & Pierce (2019), who produced 60 time-scaled phylogenetic trees. A majority-rule consensus tree was computed with branch lengths from these 60 trees using the consensus edges function of the *phytools* package (Revell, 2012) in R version 4.2.2 (R Development Core Team, 2022).

To account for the phylogenetic relationships between taxa, we performed phylogenetic generalized least squares (PGLS) analyses using the phylolm function in the *phylolm* package (Ho & Ané, 2014) in R version 4.2.2 (R Development Core Team, 2022). To analyze the dentary area relative to the lower jaw, the dentary area was included as the response variable, and the total area of lower jaw was included as an explanatory variable in the PGLS regression model (model formula: dentary area [log] ∼lower jaw area [log]). Individual PGLS regression models were built to analyze Dentary Length 1 and Dentary Length 2 relative to the lower jaw, wherein one of the two was included as the response variable, and the total length of the lower jaw was included as the explanatory variable (model formula: Dentary Length 1 [log] ∼lower jaw length [log]; and Dentary Length 2 [log] ∼lower jaw length [log]).

The ancestral states of the relative contribution of the dentary to the lower jaw were reconstructed using parsimony methods in Mesquite version 3.61 (Maddison & Maddison, 2019). To obtain univariate trait values to be used in ancestral state reconstructions, a residual value for each taxon was calculated using the PGLS regression models. A larger residual value represents a relatively larger contribution of the dentary to the lower jaw; therefore the residual values were used to quantify the relative dentary area and relative Dentary Lengths 1 and 2. These PGLS regressions were performed assuming a standard BM model of trait evolution. The statistics of these regressions are presented in Table S1.

To test the directional trend of evolution of the relative contribution of the dentary to the lower jaw, we used a BM model with a trend (trend model) for trait evolution implemented in the phylolm function in the *phylolm* package (Ho & Ané, 2014) in R version 4.2.2 (R Development Core Team, 2022). Using this approach, the trend parameter, which represents the distance from the root of the phylogenetic tree, is added to the PGLS regression models wherein a BM model was assumed for trait evolution; this allows for the estimation of this parameter. If a directional trend is present, taxa that have diverged further from the root should change more in a given direction (Pagel, 1999; Pagel, 2002).

For the PGLS regression models, we additionally fitted eight models of trait evolution that are implemented in the phylolm function in the *phylolm* package (Ho & Ané, 2014) in R version 4.2.2 (R Development Core Team, 2022) and compared support for these models using the Akaike information criterion (AIC). These models included BM, Ornstein–Uhlenbeck process with an ancestral state to be estimated at the root (OU fixed root), Ornstein–Uhlenbeck process with the ancestral state at the root having stationary distribution (OU random root), Pagel’s lambda (lambda), Pagel’s kappa (kappa), Pagel’s delta (delta), early burst (EB), and trend. The model with the lowest AIC value was selected as the best-supported model. Models with AIC differences (ΔAIC) of <2 were considered equally supported (Burnham & Anderson, 2002).

## Results

**Figure 2:**
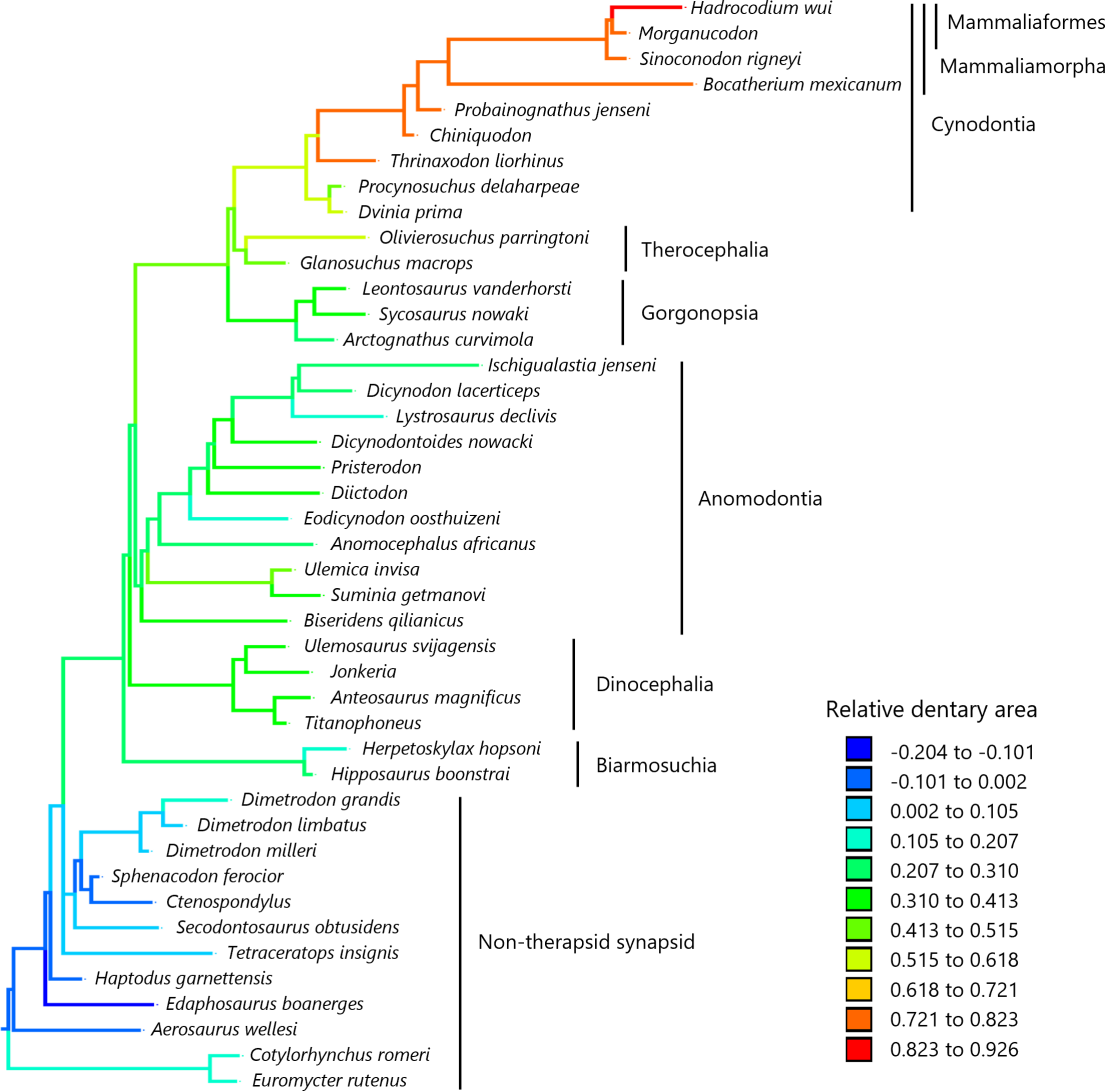
Evolutionary history of the dentary area relative to the lower jaw. The relative dentary area was calculated as a residual from the phylogenetic generalized least squares (PGLS) regression of the dentary area on the lower jaw area. Regression statistics are provided in Table S1A. Branches are colored according to the ancestral states reconstructed using parsimony methods. The values of the ancestral states at each node, which is numbered in Fig. S1, can be found in Table S2.

**Figure 3:**
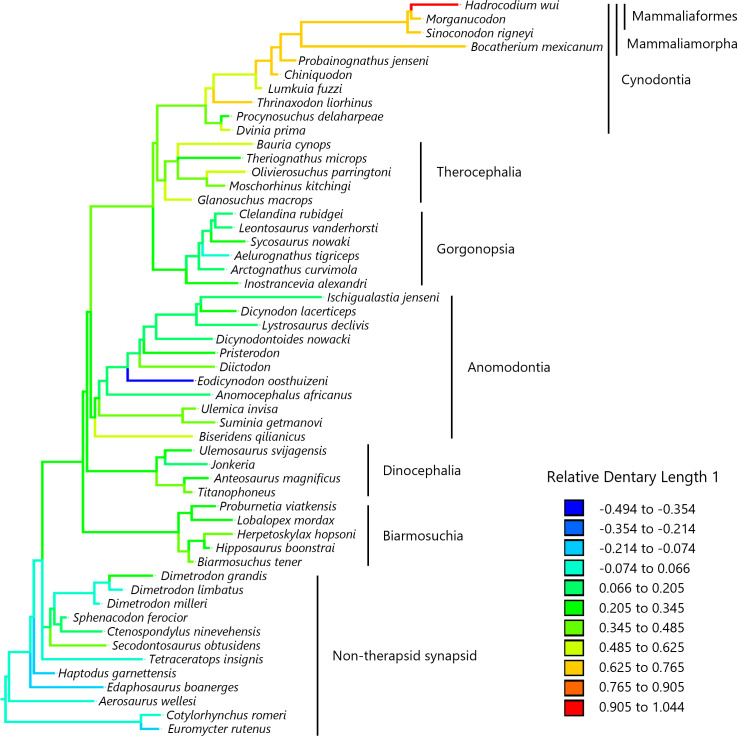
Evolutionary history of Dentary Length 1 relative to the lower jaw. Relative Dentary Length 1 was calculated as a residual from the PGLS regression of Dentary Length 1 on the lower jaw length (see Fig. 1 for definitions of length measurements). Regression statistics are provided in Table S1B. Branches are colored according to the ancestral states reconstructed using parsimony methods. Values of the ancestral states at each node, which is numbered in Fig. S2, can be found in Table S3.

**Figure 4:**
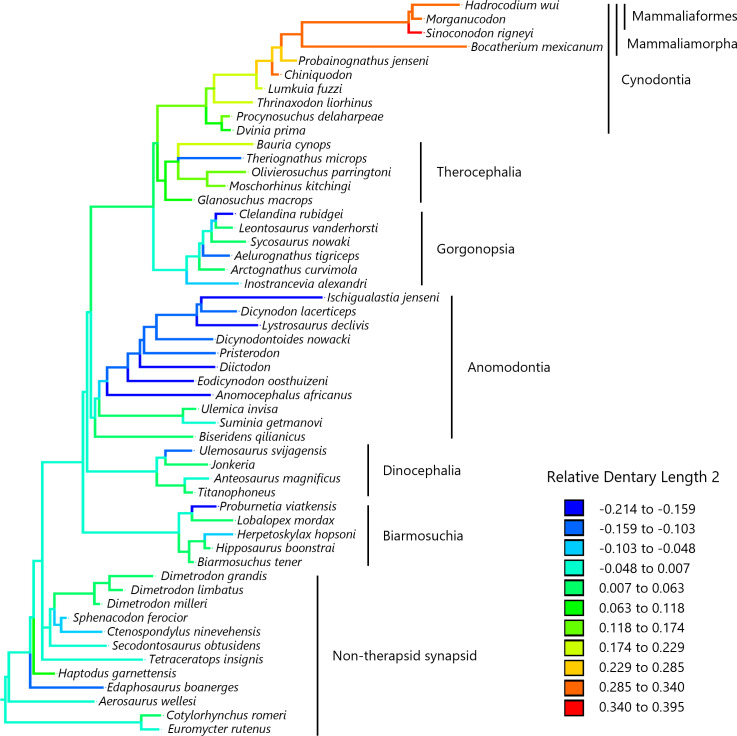
Evolutionary history of Dentary Length 2 relative to the lower jaw. Relative Dentary Length 2 was calculated as a residual from the PGLS regression of Dentary Length 2 on the lower jaw length (see Fig. 1 for definitions of length measurements). Regression statistics are provided in Table S1C. Branches are colored according to the ancestral states reconstructed using parsimony methods. Values of the ancestral states at each node, which is numbered in Fig. S3, can be found in Table S4.

To visualize the evolutionary history of the relative contribution of the dentary to the lower jaw, the reconstructed ancestral states were mapped onto phylogenetic trees (Fig. 2, relative dentary area; Fig. 3, relative Dentary Length 1; Fig. 4, relative Dentary Length 2). The relative dentary area was smallest in the earliest, non-therapsid synapsids and tended to be larger in non-cynodont therapsids and even larger in non-mammalian cynodonts; it was the largest in mammaliamorphs, especially mammaliaforms (Fig. 2). Less pronounced but generally similar differences among the synapsid taxa were observed in the relative Dentary Length 1 (Fig. 3). Relative Dentary Length 2 was largest in the mammaliamorphs but was smaller in several taxa of therapsids than in the earliest, non-therapsid synapsids (Fig. 4).

Applying a trend model in the PGLS regression of the dentary area on the lower jaw area revealed a significant effect of trend: the dentary area relative to the lower jaw increased over time across non-mammalian synapsids (Table 1). In this regression, whether assuming the trend (Table 1) or BM model (Table S1), the coefficient of the lower jaw area was estimated to be very close to 1, indicating an almost isometric relationship between the dentary and lower jaw areas. Among the eight models of trait evolution fitted in the PGLS regression, the trend model was best supported for the evolution of the dentary area relative to the lower jaw (Table 2).

When a trend model was applied in the PGLS regression of the Dentary Length 1 on the lower jaw length, the effect of trend was marginal but not significant (Table 3A). Applying a trend model in the PGLS regression of the Dentary Length 2 on the lower jaw length revealed no significant effects of trend (Table 3B). These results were qualitatively unchanged when the analysis was repeated while excluding the taxa lacking data on the dentary area (Table S5). This confirmed that the difference in the effect of trend between the analyses of the dentary area and dentary length was not because of the number of taxa in the analyses. In these regressions, regardless of whether the trend model (Table 3) or the BM model (Table S1) was used, the coefficients of the lower jaw length were estimated to be close to 1, indicating that Dentary Lengths 1 and 2 were almost isometric with respect to lower jaw length. Among the eight models of trait evolution fitted in the PGLS regressions, the trend and BM models were equally better supported for the evolution of Dentary Length 1 relative to lower jaw length (Table 4A). The lambda (estimated lambda = 0.804) and kappa (estimated kappa = 0.341) models were equally better supported for the evolution of Dentary Length 2 relative to lower jaw length (Table 4B). In the lambda model, a lambda of 1 indicates that trait evolution corresponds to a standard BM model, whereas a value close to 0 indicates that the phylogenetic signal, *i.e.,* the extent to which trait values are statistically related to phylogeny, is low (Symonds & Blomberg, 2014). In the kappa model, a kappa value of 1 indicates that trait evolution corresponds to a standard BM model, whereas a value of <1 indicates that the amount of trait change is positively correlated with the number of cladogenetic events (Pagel, 1999; Pagel, 2002).

## Discussion

In the reconstructed evolutionary history of the dentary area relative to the lower jaw in the lateral view on the phylogeny of non-mammalian synapsids, the relative dentary area appears to have gradually increased since the common ancestor of synapsids, with the largest relative dentary area observed in mammaliaforms (Fig. 2). Our analyses revealed an evolutionary trend of the relative dentary area when controlled for the effect of phylogenetic relatedness among taxa (Table 1). Furthermore, the evolution of this trait was better explained by the trend model than by the other models tested in this study (Table 2). These results demonstrated that the relative dentary area had increased over time across non-mammalian synapsids, which is consistent with the previous findings of Sidor (2003).

**Table 1:** Estimates of a phylogenetic generalized least squares (PGLS) regression of the dentary area on the lower jaw area in which a trend model was assumed for trait evolution.

Explanatory variable	Estimate	SE	*t*	*P*
Intercept	−0.967	0.127	−7.620	<0.001
Lower jaw area	0.998	0.013	75.792	<0.001
Trend	0.005	0.002	3.246	0.002

**Notes.**

The dentary area (log) was included as the response variable and the lower jaw area (log) was included as the explanatory variable.

**Table 2:** Relative supports for models of trait evolution fitted in a PGLS regression of the dentary area on the lower jaw area based on the Akaike information criterion (AIC).

Model	Log likelihood	AIC	ΔAIC
trend	−66.8	37.4	
kappa	−61.48	34.74	5.32
EB	−60.8	34.4	6
BM	−58.75	32.37	8.05
delta	−58.56	33.28	8.24
lambda	−56.75	32.37	10.05
OU fixed root	−56.75	32.37	10.05
OU random root	−54.08	31.04	12.72

**Notes.**

The dentary area (log) was included as the response variable and the lower jaw area (log) was included as the explanatory variable. ΔAIC denotes the difference in AIC values from the model with the lowest AIC.

Abbreviations BM Brownian motion OU fixed root Ornstein–Uhlenbeck process with an ancestral state to be estimated at the root OU random root Ornstein–Uhlenbeck process with the ancestral state at the root having stationary distribution lambda Pagel’s lambda kappa Pagel’s kappa delta Pagel’s delta EB early burst trend BM with a trend

**Table 3:** Estimates of PGLS regressions of the dentary length on the lower jaw length in which a trend model was assumed for trait evolution.

Explanatory variable	Estimate	SE	*t*	*P*
**(A) Dentary Length 1**				
Intercept	−1.071	0.240	−4.458	<0.001
Lower jaw length	0.998	0.041	24.429	<0.001
Trend	0.005	0.003	1.896	0.064
**(B) Dentary Length 2**				
Intercept	−0.377	0.139	−2.707	<0.001
Lower jaw length	1.018	0.024	42.913	<0.001
Trend	0.000	0.002	−0.166	0.869

**Notes.**

(A) The Dentary Length 1 (log) was included as the response variable and the lower jaw length (log) was included as the explanatory variable. (B) The Dentary Length 2 (log) was included as the response variable and the lower jaw length (log) was included as the explanatory variable (see Fig. 1 for definitions of length measurements).

**Table 4:** Relative supports for models of trait evolution fitted in a PGLS regression of the dentary length on the lower jaw length based on AIC.

Model	Log likelihood	AIC	ΔAIC
**(A) Dentary Length 1**			
trend	−29.11	18.56	
BM	−27.43	16.72	1.68
lambda	−26.92	17.46	2.19
kappa	−26.21	17.1	2.9
delta	−25.57	16.78	3.54
EB	−25.51	16.76	3.6
OU fixed root	−25.43	16.72	3.68
OU random root	−24.35	16.17	4.76
**(B) Dentary Length 2**			
lambda	−92.96	50.48	
kappa	−92.51	50.26	0.45
BM	−88.77	47.39	4.19
delta	−88.19	48.09	4.77
OU fixed root	−87.57	47.78	5.39
trend	−86.8	47.4	6.16
EB	−86.77	47.39	6.19
OU random root	−85.56	46.78	7.4

**Notes.**

(A) The Dentary Length 1 (log) was included as the response variable and the lower jaw length (log) was included as the explanatory variable. (B) The Dentary Length 2 (log) was included as the response variable and the lower jaw length (log) was included as the explanatory variable (see Fig. 1 for definitions of length measurements). ΔAIC denotes the difference in AIC values from the model with the lowest AIC.

Abbreviations BM Brownian motion OU fixed root Ornstein–Uhlenbeck process with an ancestral state to be estimated at the root OU random root Ornstein–Uhlenbeck process with the ancestral state at the root having stationary distribution lambda Pagel’s lambda kappa Pagel’s kappa delta Pagel’s delta EB early burst trend BM with a trend

Our analyses revealed no significant effect of evolutionary trend for two measurements of the dentary length relative to the lower jaw in the lateral view (Table 3). The evolution of Dentary Length 1 (see Fig. 1 for definitions of length measurements) relative to the lower jaw was equally well explained by the trend model as well as the BM model (Table 4A). The evolution of Dentary Length 2 relative to the lower jaw was better explained by the lambda and kappa models than by the other evolutionary models tested in this study (Table 4B), suggesting that the evolutionary pattern of this trait differed from the BM model of gradual evolution (Symonds & Blomberg, 2014). This kappa model, with the estimated kappa closer to zero than one, can be interpreted as a pattern closer to the punctuational mode of evolution wherein the trait changes during cladogenetic events are followed by a longer period of stasis (Pagel, 1999; Pagel, 2002). However, the phylogeny used in this study included a limited number of taxa, which may have led to a bias in the number of cladogenetic events represented in the phylogeny among different lineages and geological periods. The longest relative Dentary Lengths 1 (Fig. 3) and 2 (Fig. 4) were observed in mammaliaforms, although the reconstructed evolutionary histories indicated that these relative lengths were shorter in a number of lineages than those observed in the immediate ancestor. Therefore, the evolution of dentary enlargement at the expense of postdentary bones was not unidirectional in non-mammalian synapsids.

A directional evolutionary trend across a phylogeny can be generated by long-term natural selection (Simpson, 1944). Alternatively, this trend may be caused by developmental pathways that produce a certain type of variant rather than other types (Futuyma, 1998). This study showed an evolutionary trend toward dentary area enlargement relative to the lower jaw in the lateral view, whereas such a trend was not evident for the dentary length relative to the lower jaw in the lateral view across non-mammalian synapsids. Therefore, the evolutionary trend toward relative dentary area enlargement is likely due to the vertical expansion of the dentary rather than by reduction in the postdentary region in the lower jaw. The shape of the lower jaw varied considerably among the synapsid taxa (Sidor, 2003; Angielczyk & Kammerer, 2018; Navarro-Díaz, Esteve-Altava & Rasskin-Gutman, 2019). The height of the anterior part of the dentary increases in therapsids, like Dinocephalia and Anomodontia, more so than in early non-therapsid synapsids (Sidor, 2003). Later synapsids, especially cynodonts, also show a more developed coronoid process than their earlier relatives (Sidor, 2003; Angielczyk & Kammerer, 2018; Navarro-Díaz, Esteve-Altava & Rasskin-Gutman, 2019). These morphological changes could increase the dentary area in the lateral view of the lower jaw. They resulted in an increase in the muscle attachment area in the lower jaw (Kemp, 2005; Lautenschlager et al., 2017; Lautenschlager et al., 2018; Navarro-Díaz, Esteve-Altava & Rasskin-Gutman, 2019). Selection for increased bite force may have continually acted on the lower jaw of non-mammalian synapsids, which could be the cause of the evolutionary trend toward the vertical expansion of the dentary.

Non-mammalian synapsids showed substantial variations in body size (Sidor, 2003; Kemp, 2005). A rapid increase in body size occurred independently in two lineages of non-therapsid synapsid, Edaphosauridae and Sphenacodontia (Brocklehurst & Brink, 2017). Body size persistently reduced from early therapsids to mammaliaforms (Sookias, Butler & Benson, 2012) and was extremely miniaturized in the mammaliaforms closely related to early mammals (Lautenschlager et al., 2018). Miniaturization is considered an important factor in the evolution of thermoregulation, nocturnality, dietary ecology, and jaw morphology in early mammals (Kemp, 2005; Lovegrove, 2017; Lautenschlager et al., 2018). In the mammaliaforms, the lower jaw was almost exclusively occupied by the dentary (Luo, Schultz & Ekdale, 2016; Navarro-Díaz, Esteve-Altava & Rasskin-Gutman, 2019). Our phylogenetic regression analyses revealed that the dentary scales almost isometrically with overall lower jaw size across non-mammalian synapsids, thus supporting the previous findings of Sidor (2003). This indicates that size reduction was not a factor in the evolution of the relatively larger dentary in non-mammalian synapsids.

The gradual acquisition of some mammalian features is documented in the fossil records of synapsids (Sidor & Hopson, 1998; Rubidge & Sidor, 2001). Sidor (2001) observed an evolutionary trend toward reduction in the number of skull bones across synapsids and various synapsid subgroups. The transition from early synapsids to mammals is characterized by dentary enlargement along with reduction and eventual loss of the postdentary bones in the lower jaw (Barghusen & Hopson, 1970; Feldhamer et al., 2003; Sidor, 2003; Kemp, 2005; Kemp, 2016; Angielczyk & Kammerer, 2018; Navarro-Díaz, Esteve-Altava & Rasskin-Gutman, 2019). Recent comparative studies based on phylogeny, including that of non-mammalian synapsids, have contributed to the elucidation of the evolutionary origins and processes of mammalian features, such as endothermy (Faure-Brac & Cubo, 2020), nocturnality (Angielczyk & Schmitz, 2014), and morphologically differentiated vertebrae (Jones et al., 2018). Our phylogenetic comparative study provides insights into the evolutionary origins and processes of a series of unique characteristics of mammals, including the single-bone lower jaw, three-ossicle middle ear, and dentary–squamosal jaw joint. Our results imply that the evolutionary origin of the mammalian lower jaw is not sufficiently explained by an evolutionary trend toward dentary enlargement throughout non-mammalian synapsids. This suggests selection favoring the formation of the mammalian lower jaw during the evolution of early mammals from non-mammalian cynodonts. Selection for increased sensitivity to high-frequency sounds by reducing the postdentary bones is a possible mechanism underlying the evolution of the mammalian lower jaw (Allin, 1975; Luo, 2011; Kemp, 2016; Luo, Schultz & Ekdale, 2016). This selection may have operated exclusively in the lineage leading to mammals within cynodonts. The single-bone lower jaw and the accompanying novel dentary–squamosal jaw joint strengthen the structure against increased bite forces and provide resistance to the dislocation of the lower jaw during biting (Kemp, 2006; Tucker, 2017; Navarro-Díaz, Esteve-Altava & Rasskin-Gutman, 2019). Therefore, selection for improved efficiency of food acquisition and processing may have played a major role in the evolution of the mammalian lower jaw.

## Conclusions

We examined an evolutionary trend toward dentary enlargement and postdentary reduction in non-mammalian synapsids through phylogenetic comparative analyses. Our findings indicate that there has been an evolutionary trend for increasing the relative contribution of the dentary to the lower jaw through time across non-mammalian synapsids, regardless of their phylogenetic relatedness to mammals. This trend may be due primarily to the vertical expansion of the dentary. We found no evidence for such an evolutionary trend in dentary enlargement at the expense of the postdentary bones across non-mammalian synapsids. Thus, an evolutionary trend toward dentary enlargement across synapsids seems not to have resulted in the formation of the mammalian lower jaw, which exclusively is composed of the dentary. Instead, certain selective pressures that facilitated the evolution of the mammalian lower jaw may have arisen during the transition from non-mammalian cynodonts to early mammals.

##  Supplemental Information

10.7717/peerj.15575/supp-1 Supplemental Information 1 Estimates from phylogenetic generalized least squares (PGLS) models used to quantify the relative contribution of the dentary to the lower jaw for the ancestral state reconstructions in non-mammalian synapsids PGLS models were performed assuming a standard Brownian motion (BM) model of trait evolution. (A) The relative dentary area was calculated as a residual from the PGLS regression of the dentary area (log) on the lower jaw area (log). (B) The relative Dentary Length 1 was calculated as a residual from the PGLS regression of the Dentary Length 1 (log) on the lower jaw length (log). (C) The relative Dentary Length 2 was calculated as a residual from the PGLS regression of the Dentary Length 2 (log) on the lower jaw length (log) (see Fig. 1 for definitions of positions and length measurements).

10.7717/peerj.15575/supp-2 Supplemental Information 2 Reconstructed ancestral states of the dentary area relative to the lower jaw at each node, which is numbered in Fig. S1

10.7717/peerj.15575/supp-3 Supplemental Information 3 Reconstructed ancestral states of Dentary Length 1 relative to the lower jaw at each node, which is numbered in Fig. S2

10.7717/peerj.15575/supp-4 Supplemental Information 4 Reconstructed ancestral states of Dentary Length 2 relative to the lower jaw at each node, which is numbered in Fig. S3

10.7717/peerj.15575/supp-5 Supplemental Information 5 Estimates from PGLS regressions of the dentary length on the lower jaw length in which a trend model was assumed for trait evolution when the taxa lacking data on the dentary area were excluded from the analyses (A) The Dentary Length 1 (log) was included as the response variable and the lower jaw length (log) and the trend were included as explanatory variables. (B) The Dentary Length 2 (log) was included as the response variable and the lower jaw length (log) and the trend were included as explanatory variables (see Fig. 1 for definitions of length measurements).

10.7717/peerj.15575/supp-6 Supplemental Information 6 Positions of the numbered nodes on the phylogenetic tree of non-mammalian synapsids, which is identical to Fig. 2 The node numbers correspond to those of Table S1.

10.7717/peerj.15575/supp-7 Supplemental Information 7 Positions of the numbered nodes on the phylogenetic tree of non-mammalian synapsids, which is identical to Fig. 3 The node numbers correspond to those of Table S2.

10.7717/peerj.15575/supp-8 Supplemental Information 8 Positions of the numbered nodes on the phylogenetic tree of non-mammalian synapsids, which is identical to Fig. 4 The node numbers correspond to those of Table S3.

10.7717/peerj.15575/supp-9 Supplemental Information 9 List of the references for illustrations of the lower jaw of synapsid taxa used in this study Definitions of length measurements are shown in Fig. 1.

10.7717/peerj.15575/supp-10 Supplemental Information 10 Data on areas and lengths of the dentary and lower jaw for synapsid taxa used in this study

## Additional Information and Declarations

### Data Availability

The following information was supplied regarding data availability: The measurement data is available in the Supplementary File.

## References

[ref-1] Allin EF (1975). Evolution of the mammalian middle ear. Journal of Morphology.

[ref-2] Angielczyk KD, Kammerer CF, Zachos FE, Asher RJ (2018). Non-mammalian synapsids: the deep roots of the mammalian family tree. Handbook of zoology: mammalia: mammalian evolution, diversity and systematics.

[ref-3] Angielczyk KD, Schmitz L (2014). Nocturnality in synapsids predates the origin of mammals by over 100 million years. Proceedings of the Royal Society B: Biological Sciences.

[ref-4] Barghusen HR, Hopson JA (1970). Dentary-squamosal joint and the origin of mammals. Science.

[ref-5] Brocklehurst N, Brink KS (2017). Selection towards larger body size in both herbivorous and carnivorous synapsids during the Carboniferous. Facets.

[ref-6] Broom R (1904). On the structure of the theriodont mandible, and on its mode of articulation with the skull. Proceedings of the Zoological Society of London.

[ref-7] Burnham KP, Anderson DR (2002). Model selection and multimodel inference: a practical information-theoretic approach.

[ref-8] Church SH, Donoughe S, de Medeiros BA, Extavour CG (2019). A dataset of egg size and shape from more than 6,700 insect species. Scientific Data.

[ref-9] Faure-Brac MG, Cubo J (2020). Were the synapsids primitively endotherms? A palaeohistological approach using phylogenetic eigenvector maps. Philosophical Transactions of the Royal Society B: Biological Sciences.

[ref-10] Feldhamer GA, Drickamer LC, Vessey SH, Merritt JF (2003). Mammalogy: adaptation, diversity, and ecology.

[ref-11] Felsenstein J (1985). Phylogenies and the comparative method. The American Naturalist.

[ref-12] Futuyma DJ (1998). Evolutionary biology.

[ref-13] Harano T, Asahara M (2022). The anteriorization of tooth position underlies the atavism of tooth morphology: insights into the morphogenesis of mammalian molars. Evolution.

[ref-14] Ho LST, Ané C (2014). A linear-time algorithm for Gaussian and non-Gaussian trait evolution models. Systematic Biology.

[ref-15] Jones KE, Angielczyk KD, Pierce SE (2019). Stepwise shifts underlie evolutionary trends in morphological complexity of the mammalian vertebral column. Nature Communications.

[ref-16] Jones K, Angielczyk K, Polly P, Head JJ, Fernandez V, Lungmus JK, Tulga S, Pierce SE (2018). Fossils reveal the complex evolutionary history of the mammalian regionalized spine. Science.

[ref-17] Jones KE, Dickson BV, Angielczyk KD, Pierce SE (2021). Adaptive landscapes challenge the lateral-to-sagittal paradigm for mammalian vertebral evolution. Current Biology.

[ref-18] Kammerer CF (2016). Systematics of the Rubidgeinae (Therapsida: Gorgonopsia). PeerJ.

[ref-19] Kemp TS (2005). The origin and evolution of mammals.

[ref-20] Kemp TS (2006). The origin and early radiation of the therapsid mammal-like reptiles: a palaeobiological hypothesis. Journal of Evolutionary Biology.

[ref-21] Kemp TS, Clack JA, Fay RR, Popper AN (2016). Non-mammalian synapsids: the beginning of the mammal line. Evolution of the vertebrate ear–evidence from the fossil record.

[ref-22] Lautenschlager S, Gill P, Luo ZX, Fagan MJ, Rayfield EJ (2017). Morphological evolution of the mammalian jaw adductor complex. Biological Reviews.

[ref-23] Lautenschlager S, Gill PG, Luo ZX, Fagan MJ, Rayfield EJ (2018). The role of miniaturization in the evolution of the mammalian jaw and middle ear. Nature.

[ref-24] Lovegrove BG (2017). A phenology of the evolution of endothermy in birds and mammals. Biological Reviews.

[ref-25] Luo Z-X (2011). Developmental patterns in Mesozoic evolution of mammal ears. Annual Review of Ecology, Evolution and Systematics.

[ref-26] Luo Z-X, Schultz JA, Ekdale EG, Clack JA, Fay RR, Popper AN (2016). Evolution of the middle and inner ears of mammaliaforms: the approach to mammals. Evolution of the vertebrate ear–evidence from the fossil record.

[ref-27] Maddison WP, Maddison DR (2019).

[ref-28] Navarro-Díaz A, Esteve-Altava B, Rasskin-Gutman D (2019). Disconnecting bones within the jaw-otic network modules underlies mammalian middle ear evolution. Journal of Anatomy.

[ref-29] Olson EC (1944). Origin of mammals based upon cranial morphology of the therapsid suborders. Geological Society of America Special Papers.

[ref-30] Pagel M (1999). Inferring the historical patterns of biological evolution. Nature.

[ref-31] Pagel M, MacLeod N, Forey PL (2002). Modelling the evolution of continuously varying characters on phylogenetic trees: the case of hominid cranial capacity. Morphology, shape and phylogeny.

[ref-32] R Development Core Team (2022). R: a language and environment for statistical computing.

[ref-33] Revell LJ (2012). phytools: an R package for phylogenetic comparative biology (and other things). Methods in Ecology and Evolution.

[ref-34] Rohlf FJ (2017). tpsDig, digitize landmarks and outlines.

[ref-35] Rowe T (1988). Definition, diagnosis, and origin of Mammalia. Journal of Vertebrate Paleontology.

[ref-36] Rubidge BS, Sidor CA (2001). Evolutionary patterns among Permo-Triassic therapsids. Annual Review of Ecology and Systematics.

[ref-37] Schneider CA, Rasband W, Eliceiri KW (2012). NIH Image to ImageJ: 25 years of image analysis. Nature Methods.

[ref-38] Sidor CA (2001). Simplification as a trend in synapsid cranial evolution. Evolution.

[ref-39] Sidor CA (2003). Evolutionary trends and the origin of the mammalian lower jaw. Paleobiology.

[ref-40] Sidor CA, Hopson JA (1998). Ghost lineages and mammalness assessing the temporal pattern of character acquisition in the Synapsida. Paleobiology.

[ref-41] Simpson GG (1944). Tempo and mode in evolution.

[ref-42] Slater GJ, Van Valkenburgh B (2008). Long in the tooth: evolution of sabertooth cat cranial shape. Paleobiology.

[ref-43] Sookias RB, Butler RJ, Benson RB (2012). Rise of dinosaurs reveals major body-size transitions are driven by passive processes of trait evolution. Proceedings of the Royal Society of London Series B: Biological Sciences.

[ref-44] Symonds MRE, Blomberg SP, Garamszegi L (2014). A primer on phylogenetic generalised least squares. Modern phylogenetic comparative methods and their application in evolutionary biology.

[ref-45] Tucker AS (2017). Major evolutionary transitions and innovations: the tympanic middle ear. Philosophical Transactions of the Royal Society B: Biological Sciences.

