# Supplementary material for: Revisiting the evolutionary trend toward the mammalian lower jaw in non-mammalian synapsids in a phylogenetic context
Source: PeerJ. 2023 Jun 20;11:e15575. doi: 10.7717/peerj.15575 (PMC10289081; doi:10.7717/peerj.15575)
Supplement: Supplemental Information 1 — PGLS models were performed assuming a standard Brownian motion (BM) model of trait evolution. (A) The relative dentary area was calculated as a residual from the PGLS regression of the dentary area (log) on the lower jaw area (log). (B) The relative Dentary Length 1 was calculated as a residual from the PGLS regression of the Dentary Length 1 (log) on the lower jaw length (log). (C) The relative Dentary Length 2 was calculated as a residual from the PGLS regression of the Dentary Length 2 (log) on the lower jaw length (log) (see Fig. 1 for definitions of positions and length measurements). [file peerj-11-15575-s001.pdf]

Supplemental Information

Table S1 Estimates from phylogenetic generalized least squares (PGLS) models used to quantify the relative contribution of the dentary to the lower jaw for the ancestral state reconstructions in non-mammalian synapsids.

Explanatory variable	Estimate	SE	t	P
(A) Relative dentary area				
Intercept	-0.816	0.131	-6.226	<0.001
Lower jaw area	0.995	0.015	68.244	<0.001
(B) Relative Dentary Length 1				
Intercept	-0.907	0.230	-3.947	<0.001
Lower jaw length	0.990	0.042	23.769	<0.001
(C) Relative Dentary Length 2				
Intercept	-0.386	0.129	-2.995	<0.001
Lower jaw length	1.018	0.023	43.593	<0.001

PGLS models were performed assuming a standard Brownian motion (BM) model of trait evolution. (A) The relative dentary area was calculated as a residual from the PGLS regression of the dentary area (log) on the lower jaw area (log). (B) The relative Dentary Length 1 was calculated as a residual from the PGLS regression of the Dentary Length 1 (log) on the lower jaw length (log). (C) The relative Dentary Length 2 was calculated as a residual from the PGLS regression of the Dentary Length 2 (log) on the lower jaw length (log) (see Fig. 1 for definitions of positions and length measurements).
