# Supplementary material for: Revisiting the evolutionary trend toward the mammalian lower jaw in non-mammalian synapsids in a phylogenetic context
Source: PeerJ. 2023 Jun 20;11:e15575. doi: 10.7717/peerj.15575 (PMC10289081; doi:10.7717/peerj.15575)
Supplement: Supplemental Information 2 [file peerj-11-15575-s002.pdf]

Supplemental Information

Table S2: Reconstructed ancestral states of the dentary area relative to the lower jaw at each node, which is numbered in Fig. S1.

Node	State at ancestor	State at node
3	-0.000002	-0.005325
4	-0.005325	-0.024700
5	-0.024700	-0.018373
6	-0.018373	0.015044
7	0.015044	0.238365
8	0.238365	0.260565
9	0.260565	0.278204
10	0.278204	0.419660
11	0.419660	0.434876
12	0.434876	0.566074
13	0.566074	0.611453
14	0.611453	0.764883
15	0.764883	0.783157
16	0.783157	0.787222
17	0.787222	0.787051
18	0.787051	0.788029
19	0.788029	0.823346
20	0.788029	0.783679
21	0.787051	0.784096
22	0.787222	0.819598
23	0.783157	0.807506
24	0.764883	0.768877
25	0.611453	0.721820
26	0.566074	0.519992
27	0.519992	0.467192
28	0.519992	0.559061
29	0.434876	0.443147
30	0.443147	0.522522
31	0.443147	0.443506

32	0.419660	0.352570
33	0.352570	0.365594
34	0.365594	0.401791
35	0.365594	0.341319
36	0.352570	0.290097
37	0.278204	0.286613
38	0.286613	0.290577
39	0.290577	0.286045
40	0.286045	0.284572
41	0.284572	0.305106
42	0.305106	0.309330
43	0.309330	0.316300
44	0.316300	0.285839
45	0.285839	0.288853
46	0.288853	0.309821
47	0.288853	0.308568
48	0.285839	0.197018
49	0.316300	0.390150
50	0.309330	0.342294
51	0.305106	0.352776
52	0.284572	0.170881
53	0.286045	0.236577
54	0.290577	0.418943
55	0.418943	0.453936
56	0.418943	0.374274
57	0.286613	0.392143
58	0.260565	0.338748
59	0.338748	0.336893
60	0.336893	0.334532
61	0.336893	0.331829
62	0.338748	0.376078
63	0.376078	0.311238
64	0.376078	0.403684
65	0.238365	0.245057
66	0.245057	0.178997
67	0.245057	0.256100
68	0.015044	0.001891

69	0.001891	-0.010663
70	-0.010663	0.036496
71	0.036496	0.076512
72	0.076512	0.181196
73	0.076512	0.077032
74	0.036496	0.030552
75	-0.010663	-0.039959
76	-0.039959	-0.056017
77	-0.039959	-0.052978
78	0.001891	0.082969
79	0.015044	0.036139
80	-0.018373	-0.068094
81	-0.024700	-0.203505
82	-0.005325	-0.037226
83	-0.000002	0.177717
84	0.177717	0.204952
85	0.177717	0.175131
