# Supplementary material for: Revisiting the evolutionary trend toward the mammalian lower jaw in non-mammalian synapsids in a phylogenetic context
Source: PeerJ. 2023 Jun 20;11:e15575. doi: 10.7717/peerj.15575 (PMC10289081; doi:10.7717/peerj.15575)
Supplement: Supplemental Information 3 [file peerj-11-15575-s003.pdf]

Table S3: Reconstructed ancestral states of Dentary Length 1 relative to the lower jaw at each node, which is numbered in Fig. S2.

Node	State at ancestor	State at node
3	0.000011	0.000725
4	0.000725	0.001161
5	0.001161	0.005968
6	0.005968	0.064351
7	0.064351	0.227070
8	0.227070	0.238887
9	0.238887	0.246977
10	0.246977	0.368072
11	0.368072	0.391975
12	0.391975	0.468007
13	0.468007	0.513335
14	0.513335	0.603403
15	0.603403	0.674703
16	0.674703	0.679960
17	0.679960	0.688935
18	0.688935	0.711293
19	0.711293	0.706582
20	0.706582	0.904600
21	0.706582	0.661800
22	0.711293	0.728000
23	0.688935	0.725500
24	0.679960	0.681600
25	0.674703	0.697000
26	0.603403	0.592000
27	0.513335	0.643600
28	0.468007	0.403531
29	0.403531	0.276500
30	0.403531	0.520400
31	0.391975	0.426938
32	0.426938	0.437489

33	0.437489	0.552900
34	0.437489	0.315200
35	0.437489	0.474265
36	0.474265	0.561000
37	0.474265	0.457000
38	0.426938	0.512700
39	0.368072	0.236726
40	0.236726	0.167761
41	0.167761	0.150437
42	0.150437	0.157505
43	0.157505	0.149217
44	0.149217	0.119300
45	0.149217	0.146600
46	0.157505	0.253300
47	0.150437	0.020200
48	0.167761	0.133500
49	0.236726	0.323600
50	0.246977	0.247165
51	0.247165	0.236290
52	0.236290	0.199817
53	0.199817	0.108419
54	0.108419	0.167006
55	0.167006	0.173950
56	0.173950	0.168682
57	0.168682	0.176337
58	0.176337	0.180919
59	0.180919	0.104200
60	0.180919	0.242300
61	0.176337	0.120400
62	0.168682	0.134000
63	0.173950	0.324800
64	0.167006	0.396000
65	0.108419	-0.493700
66	0.199817	0.200900
67	0.236290	0.387995
68	0.387995	0.410600
69	0.387995	0.384800

70	0.247165	0.509800
71	0.238887	0.302792
72	0.302792	0.294253
73	0.294253	0.330600
74	0.294253	0.194800
75	0.302792	0.356035
76	0.356035	0.322300
77	0.356035	0.378400
78	0.227070	0.333463
79	0.333463	0.299405
80	0.299405	0.216100
81	0.299405	0.340200
82	0.333463	0.370630
83	0.370630	0.325204
84	0.325204	0.386400
85	0.325204	0.302500
86	0.370630	0.397300
87	0.064351	0.090575
88	0.090575	0.084665
89	0.084665	0.024874
90	0.024874	0.064230
91	0.064230	0.248800
92	0.064230	0.041300
93	0.024874	0.007300
94	0.084665	0.085049
95	0.085049	0.085600
96	0.085049	0.081900
97	0.090575	0.348600
98	0.064351	0.058000
99	0.005968	-0.110700
100	0.001161	-0.082300
101	0.000725	0.013700
102	0.000011	-0.023817
103	-0.023817	0.035600
104	-0.023817	-0.081000
