# Supplementary material for: Revisiting the evolutionary trend toward the mammalian lower jaw in non-mammalian synapsids in a phylogenetic context
Source: PeerJ. 2023 Jun 20;11:e15575. doi: 10.7717/peerj.15575 (PMC10289081; doi:10.7717/peerj.15575)
Supplement: Supplemental Information 4 [file peerj-11-15575-s004.pdf]

Table S4: Reconstructed ancestral states of Dentary Length 2 relative to the lower jaw at each node, which is numbered in Fig. S3.

Node	State at ancestor	State at node
3	-0.000001	-0.000199
4	-0.000199	-0.000838
5	-0.000838	0.005387
6	0.005387	-0.013650
7	-0.013650	-0.024956
8	-0.024956	-0.027107
9	-0.027107	-0.029108
10	-0.029108	0.041802
11	0.041802	0.054042
12	0.054042	0.130729
13	0.130729	0.152411
14	0.152411	0.194616
15	0.194616	0.267118
16	0.267118	0.274552
17	0.274552	0.285310
18	0.285310	0.332838
19	0.332838	0.332275
20	0.332275	0.324700
21	0.332275	0.332500
22	0.332838	0.340000
23	0.285310	0.297300
24	0.274552	0.278200
25	0.267118	0.288600
26	0.194616	0.177800
27	0.152411	0.215500
28	0.130729	0.113405
29	0.113405	0.137300
30	0.113405	0.076900
31	0.054042	0.065569
32	0.065569	0.072493

33	0.072493	0.184100
34	0.072493	-0.119200
35	0.072493	0.134759
36	0.134759	0.154500
37	0.134759	0.162300
38	0.065569	0.086900
39	0.041802	-0.020691
40	-0.020691	-0.033178
41	-0.033178	-0.044924
42	-0.044924	-0.045616
43	-0.045616	-0.055813
44	-0.055813	-0.181200
45	-0.055813	0.027800
46	-0.045616	0.033600
47	-0.044924	-0.116100
48	-0.033178	0.012700
49	-0.020691	-0.063600
50	-0.029108	-0.035736
51	-0.035736	-0.046025
52	-0.046025	-0.073311
53	-0.073311	-0.113436
54	-0.113436	-0.124181
55	-0.124181	-0.125508
56	-0.125508	-0.129369
57	-0.129369	-0.152253
58	-0.152253	-0.152791
59	-0.152791	-0.208100
60	-0.152791	-0.141400
61	-0.152253	-0.177900
62	-0.129369	-0.115600
63	-0.125508	-0.126200
64	-0.124181	-0.165200
65	-0.113436	-0.184200
66	-0.073311	-0.214300
67	-0.046025	0.023068
68	0.023068	0.048000
69	0.023068	-0.017100

70	-0.035736	0.051200
71	-0.027107	-0.029667
72	-0.029667	-0.041858
73	-0.041858	-0.108800
74	-0.041858	0.007800
75	-0.029667	0.008772
76	0.008772	0.001400
77	0.008772	0.020000
78	-0.024956	-0.001070
79	-0.001070	-0.045921
80	-0.045921	-0.168400
81	-0.045921	0.030100
82	-0.001070	0.035678
83	0.035678	0.037079
84	0.037079	-0.088500
85	0.037079	0.058000
86	0.035678	0.050200
87	-0.013650	-0.030953
88	-0.030953	-0.041429
89	-0.041429	0.017806
90	0.017806	0.017628
91	0.017628	0.045800
92	0.017628	0.009400
93	0.017806	0.024400
94	-0.041429	-0.069312
95	-0.069312	-0.085100
96	-0.069312	-0.076800
97	-0.030953	-0.006200
98	-0.013650	-0.007100
99	0.005387	0.080600
100	-0.000838	-0.113100
101	-0.000199	-0.001700
102	-0.000001	0.006588
103	0.006588	0.013400
104	0.006588	0.001200
