# Supplementary material for: Revisiting the evolutionary trend toward the mammalian lower jaw in non-mammalian synapsids in a phylogenetic context
Source: PeerJ. 2023 Jun 20;11:e15575. doi: 10.7717/peerj.15575 (PMC10289081; doi:10.7717/peerj.15575)
Supplement: Supplemental Information 5 — (A) The Dentary Length 1 (log) was included as the response variable and the lower jaw length (log) and the trend were included as explanatory variables. (B) The Dentary Length 2 (log) was included as the response variable and the lower jaw length (log) and the trend were included as explanatory variables (see Fig. 1 for definitions of length measurements). [file peerj-11-15575-s005.pdf]

Table S5: Estimates from PGLS regressions of the dentary length on the lower jaw length in which a trend model was assumed for trait evolution when the taxa lacking data on the dentary area were excluded from the analyses.

Explanatory variable	Estimate	SE	t	P
(A) Dentary Length 1				
Intercept	-1.055	0.294	-3.585	<0.001
Lower jaw length	0.993	0.052	19.191	<0.001
Trend	0.006	0.003	1.893	0.066
(B) Dentary Length 2				
Intercept	-0.359	0.124	-2.885	0.006
Lower jaw length	1.013	0.022	46.332	<0.001
Trend	0.000	0.001	-0.057	0.955

(A) The Dentary Length 1 (log) was included as the response variable and the lower jaw length (log) and the trend were included as explanatory variables. (B) The Dentary Length 2 (log) was included as the response variable and the lower jaw length (log) and the trend were included as explanatory variables (see Fig. 1 for definitions of length measurements).
