# Supplementary material for: Revisiting the evolutionary trend toward the mammalian lower jaw in non-mammalian synapsids in a phylogenetic context
Source: PeerJ. 2023 Jun 20;11:e15575. doi: 10.7717/peerj.15575 (PMC10289081; doi:10.7717/peerj.15575)
Supplement: Supplemental Information 7 — The node numbers correspond to those of Table S2. [file peerj-11-15575-s007.pdf]

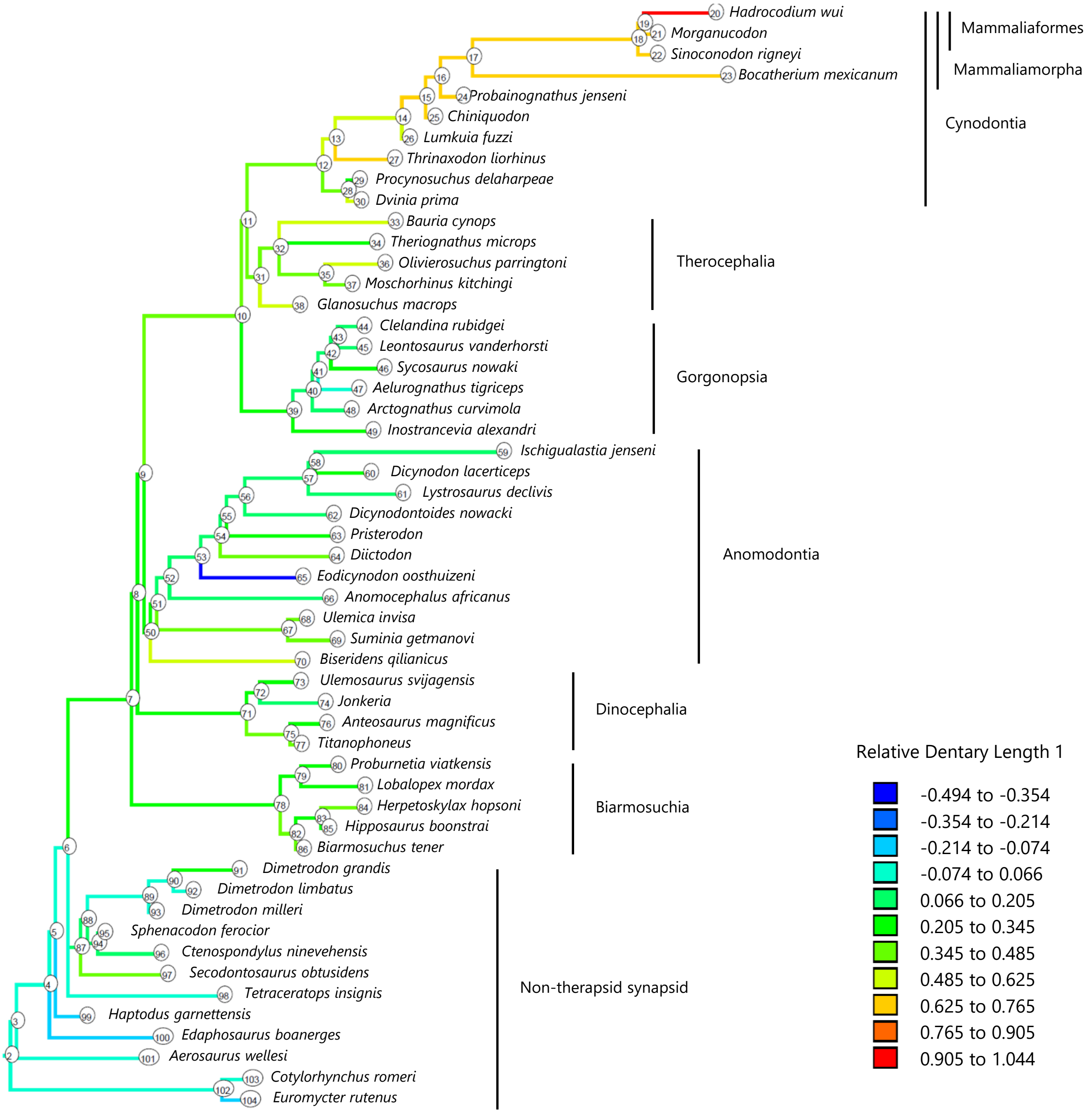

Figure S2: Positions of the numbered nodes on the phylogenetic tree of non-mammalian synapsids, which is identical to Fig. 3. The node numbers correspond to those of Table S2.
